# Anti‐nuclear matrix protein 2 antibody‐positive amyopathic dermatomyositis presenting in a patient with prostate cancer: A case report

**DOI:** 10.1002/ccr3.8884

**Published:** 2024-05-08

**Authors:** Divya M. Shan, Neha Gupta, Alex G. Ortega‐Loayza, Sofia Shea, Abhishek Nandan

**Affiliations:** ^1^ School of Medicine Virginia Commonwealth University Richmond Virginia USA; ^2^ Division of Rheumatology, Allergy and Immunology Virginia Commonwealth University School of Medicine Richmond Virginia USA; ^3^ Department of Dermatology Oregon Health & Science University Portland Oregon USA; ^4^ Dermatopathology Hunter Holmes McGuire Veterans Affairs Medical Center Richmond Virginia USA; ^5^ Rheumatology Hunter Holmes McGuire Veterans Affairs Medical Center Richmond Virginia USA

**Keywords:** amyopathic dermatomyositis, cutaneous lupus, nuclear matrix protein, NXP‐2, prostate adenocarcinoma, prostate cancer

## Abstract

Nuclear matrix protein (NXP‐2) positive amyopathic dermatomyositis (DM) may present without classic symptoms like muscle weakness, dysphagia, and edema, and mimic conditions like cutaneous lupus. Given DM's association with malignancy and interstitial lung disease, prompt and accurate diagnosis is important. Testing for myositis‐specific antibodies aids diagnosis in ambiguous cases.

## Graphical abstract

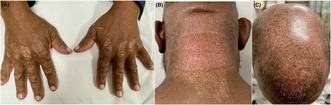

## 1 INTRODUCTION

The hallmark feature of dermatomyositis (DM), an idiopathic inflammatory disorder, is symmetric proximal skeletal muscle weakness with characteristic cutaneous manifestations.^1^ Besides the pathognomonic findings of Gottron papules and heliotrope rash, other additional skin manifestations include facial erythema, shawl sign, poikiloderma, and calcinosis cutis.^2^ There can also be associated extramuscular manifestations, such as interstitial lung disease (ILD), myocarditis, and esophageal dysphagia. DM can also be strongly associated with malignancy in adults.

Laboratory findings are typically notable for elevated muscle enzymes such as creatine kinase, aldolase, and lactate dehydrogenase, as well as disease‐specific autoantibodies.^3^ Histopathology can be a helpful diagnostic tool in these patients, with unique disease findings on muscle and skin biopsies. Characteristic muscle biopsies of DM often reveal a perivascular and perimysial inflammatory infiltrate as well as perifascicular atrophy.^4^ The skin biopsy findings in DM can be similar to that of systemic lupus erythematosus (SLE) with interface dermatitis, increased lymphocytic infiltrate, and mucin deposition in the dermis.^5^

DM is associated with several myositis specific autoantibodies that each have unique clinical phenotypes within the disease spectrum: anti‐Jo, anti‐Mi‐2, anti‐SRP, anti‐MDA5, anti‐TIF‐1 gamma, anti‐SAE, and anti‐NXP2.^6^ Individuals with anti‐nuclear matrix protein 2 (NXP‐2) antibodies are typically characterized by severe muscle weakness, calcinosis, dysphagia, and peripheral edema.^7^ Here we present an atypical case of the amyopathic NXP‐2 positive DM subtype in a 67‐year‐old male with prostate cancer lacking the classic features associated with NXP‐2 antibodies.

## 2 CASE PRESENTATION

A 67‐year‐old male with a history of well‐controlled hypertension, well‐controlled type 2 diabetes mellitus, and prostate adenocarcinoma (Gleason 3 + 3) on active surveillance without prior treatment initially presented to a dermatology clinic with an acute presentation of rash involving his scalp, face, hands, and chest. He reported seasonal allergies and frequently worked around cleaning products and chemicals. The physical exam revealed hyperkeratotic hypopigmented plaques on the dorsal hands and poikilodermatous skin changes on the posterior neck. There was also xerotic skin noted on the trunk as well as arms and legs.

## 3 METHODS

The patient's atopic background and presence of eczematous dermatitis prompted treatment with topical fluocinonide. However, the patient did not endorse clinical improvement, and further workup was pursued. Laboratory results for the patient revealed anemia and elevated antinuclear antibodies (ANA) with a titer of 1:2560 in a speckled pattern. Shave biopsies of the left dorsal thumb and posterior neck revealed interface dermatitis with neutrophil‐rich dermal inflammation and marked papillary dermal edema. The patient was given a working diagnosis of cutaneous lupus, and rheumatology was consulted for further management.

The patient presented to the rheumatology clinic without complaints of sicca symptoms, muscle weakness, oral ulcers, chest pain, Raynaud's, or joint pain and swelling. The patient reported mild, intermittent shortness of breath since an infection with the novel coronavirus in 2020. The dermatologic exam by rheumatology revealed hypopigmented plaques on the dorsal hands and posterior neck, as well as mild erythema on the forehead and cheek. There were no objective findings of muscle weakness or joint disease. The patient's laboratory results showed anemia but otherwise normal complete blood count, creatinine, liver function, and microalbumin/creatinine ratio. Additional laboratory studies were notable for positive anti‐SSA (>8 units, normal <1) while other antibodies, including anti‐dsDNA, anti‐Sm, and anti‐U1 RNP were negative. With a high positive anti‐SSA, the patient was empirically treated for SLE with a trial of hydroxychloroquine (HCQ) 200 mg twice daily.

**FIGURE 1:**
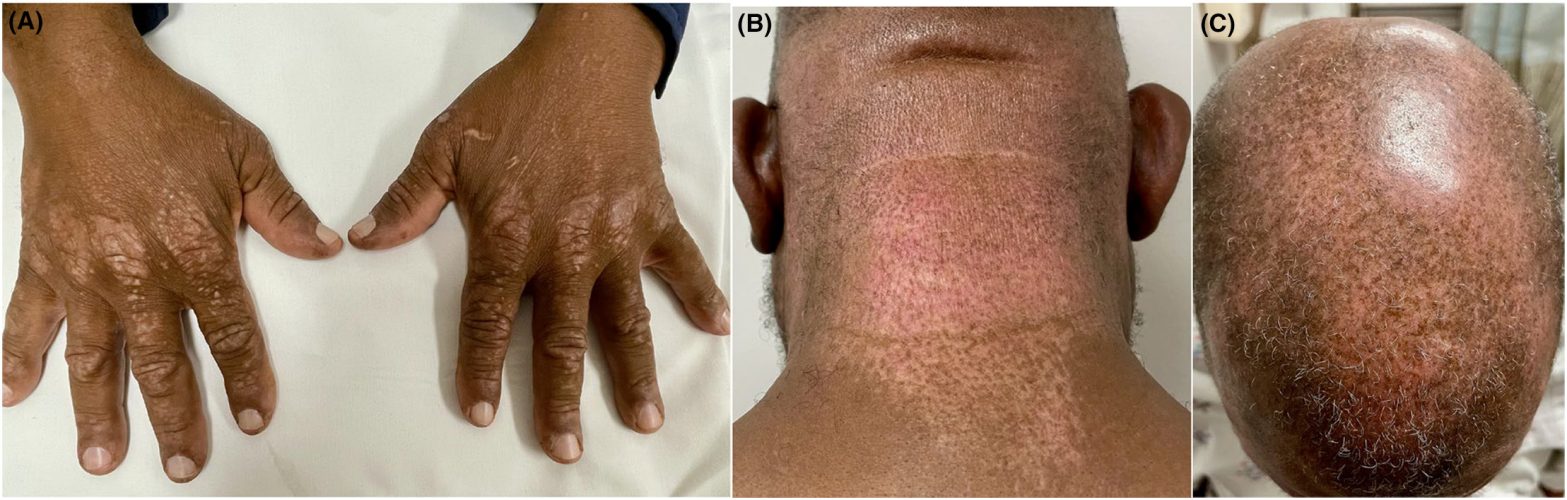
(A) Gottron papules presenting as hyperkeratotic, hypopigmented plaques on dorsal hands, (B) Poikilodermatous skin changes on the posterior neck, (C) Poikilodermatous skin changes on the scalp.

**FIGURE 2:**
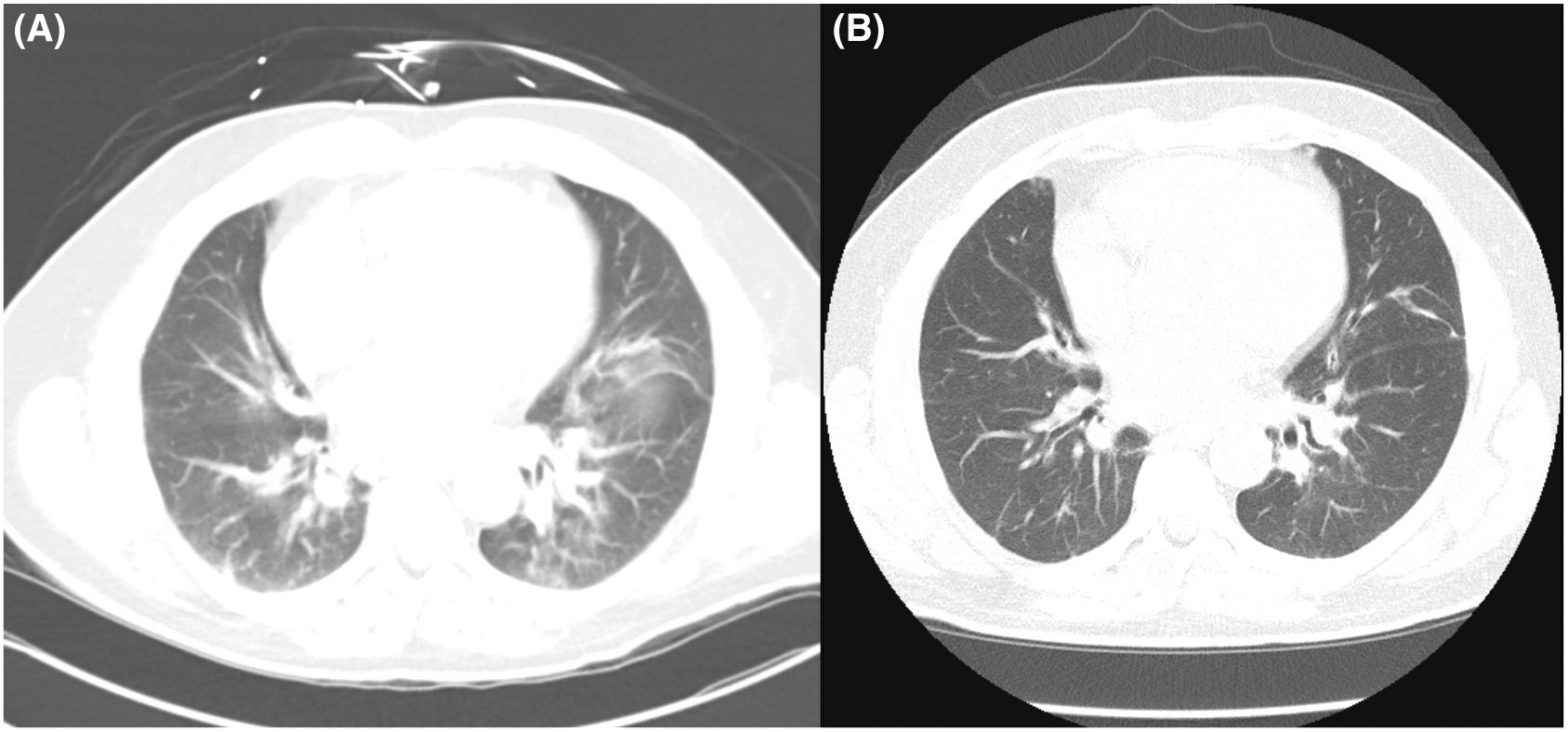
CT scan of the chest (A) before treatment and (B) after 5 months of treatment with improvement on prednisone taper and mycophenolate mofetil.

A few months later, the patient was hospitalized due to progressive dyspnea, persistent cough, and a decline in functioning. He had visited the emergency department twice and was discharged with antibiotics for treatment of pneumonia, which did not improve his symptoms. Physical exam revealed progressing hypopigmented patches on the dorsal hands, scalp, anterior chest, and neck despite daily use of HCQ (Figure 1). His infectious workup, including blood tests and bronchoalveolar lavage of the right middle and lower lobes, was negative for fungus, mycobacteria, gram stain, tuberculosis, rapid plasma reagin, hepatitis B/C, human immunodeficiency virus. Chest computed tomography (CT) scan showed bibasilar ground glass and nodular opacities that had persisted on multiple CT scans more than 8 weeks apart (Figure 2). This was concerning for ILD and suggested a possible rheumatic origin for the lung findings. Laboratory findings studies were notable for mild aspartate aminotransferase elevation without alanine transaminase elevation. His creatinine phosphokinase returned minimally elevated at 476 IU/L (normal: 39–308). A myositis panel was obtained given the clinical suspicion for autoimmune ILD in the setting of known rash with interface dermatitis on biopsy. The results were notably positive for NXP‐2 antibody, while other myositis‐specific antibodies were negative.

The patient was ultimately diagnosed with amyopathic NXP‐2 positive DM. Given the concern for amyopathic DM leading to ILD, the patient was placed on a prednisone taper and mycophenolate mofetil 1000 mg twice daily.

## 4 CONCLUSION AND RESULTS

Given that this antibody carries a high‐grade malignancy risk, the patient underwent a malignancy workup. CT abdomen/pelvis revealed stable but persistent prostatomegaly. His prostate‐specific antigen returned at 12.4 ng/mL (prior value 11.0; ref: 0–3.0). The remainder of his age‐appropriate malignancy screening returned negative.

**FIGURE 3:**
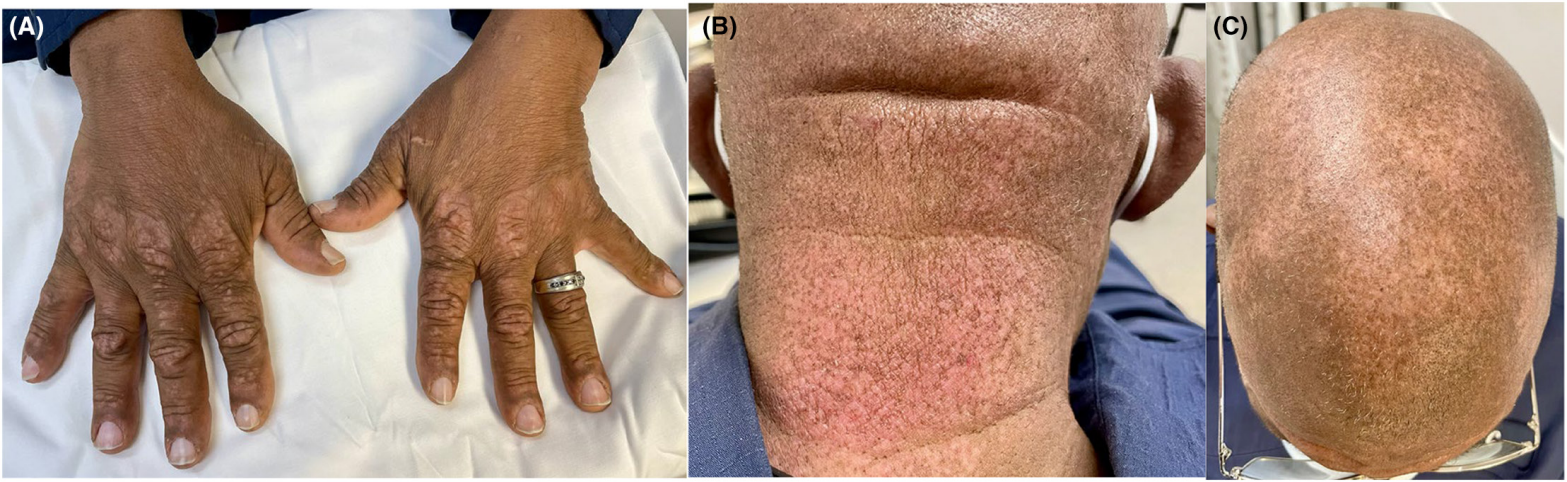
(A) Gottron papules, (B) Poikilodermatous skin changes on the posterior neck, (C) Poikilodermatous skin changes on the scalp after 5 months of treatment with prednisone taper and mycophenolate mofetil.

After 5 months of treatment, the patient had improved respiratory symptoms and the chest CT showed resolution of previously seen bibasilar ground glass opacities (Figure 2). The patient also noted mild improvement of rashes on the back of his hands, neck, and scalp with treatment (Figure 3). He continues to have close follow‐up with oncology for careful active surveillance of his low‐grade prostate adenocarcinoma.

## 5 DISCUSSION

DM is an inflammatory disorder that presents with varying degrees of muscle weakness and cutaneous manifestations, such as Gottron papules and heliotrope rash. Amyopathic DM represents a subset of 5%–20% of patients with DM who present with characteristic skin manifestations without any signs of muscle weakness or elevated muscle enzymes.^8^ However, these cutaneous manifestations can be heterogeneous and difficult to recognize, especially early in the disease course like in this case. Some studies have associated the NXP‐2 phenotype with a lack of skin findings or nonspecific skin rashes that stray from typical DM findings.^9^, ^10^, ^11^

The diagnosis of amyopathic DM can also be challenging due to overlapping features with conditions such as cutaneous lupus erythematosus. Both conditions can present with photosensitivity, elevated ANA, and indistinguishable histologic findings (e.g., interface dermatitis with dermal mucin deposition).^12^, ^13^ Histologic findings of lymphocytic infiltrate are typically associated with DM; this case highlights a less common presentation of neutrophilic infiltrate in DM.^14^ While anti‐SSA and anti‐SSB antibodies are typically found in SLE and Sjogren syndrome, they can also be found in other autoimmune diseases such as DM, rheumatoid arthritis, and systemic sclerosis.^15^ Thus, testing for myositis‐specific antibodies can be valuable in the diagnosis of atypical or ambiguous cases of inflammatory myopathies.

DM, especially with antibodies to NXP‐2, is strongly associated with an increased risk for malignancies in adults.^16^ In particular, the most commonly associated cancers were hematologic, ovarian, breast, and lung cancers.^17^ Despite prostate cancer being the most common malignancy in men (not including skin cancer), it has not been commonly associated with amyopathic DM in male patients.^18^ This case demonstrates the importance of assessing for any underlying malignancy in patients with DM. Patients with concomitant DM and malignancy have poor prognosis; therefore, early clinical recognition of this disease is important.^19^ Patients with DM are also commonly affected by concomitant ILD, which can present variably as mild or rapidly progressive. Therefore, clinicians should consider the possibility of underlying DM in patients with malignancies and ILD, even if they do not have any evidence of muscle involvement. Other manifestations of the disease include dysphagia, dysphonia, myalgias, and Raynaud phenomenon.^1^

Despite the association of the NXP‐2 antibody with severe muscle weakness, this patient did not present with muscle weakness or objective musculoskeletal findings. He also did not present with any of the other features associated with the NXP‐2 antibody such as calcinosis, dysphagia, and edema.^20^ Notably, certain features such as calcinosis are particularly characteristic of this antibody's pathological association in children.^21^ Our case adds to the current knowledge of the heterogeneous presentations of this rare DM subtype in adults.

The first‐line therapy for DM is oral prednisone, which can be slowly tapered after symptomatic relief is achieved with the addition of disease‐modifying drugs, such as methotrexate and azathioprine. Alternatives to these agents include mycophenolate mofetil, which is increasingly being used in patients with DM who also have skin disease, and tacrolimus.^22^, ^23^ Both of these agents are especially useful in cases with concomitant ILD.^23^, ^24^, ^25^ In individuals with refractory disease, therapies such as rituximab, intravenous immunoglobulin (IVIG), and other biologics can also be considered.^26^

The management of the cutaneous manifestations of DM can be challenging given that they are often more resistant to treatment than muscle involvement, and the best treatment approach is still unclear. Interventions that are recommended for all patients include photoprotection, antipruritic agents, and topical steroids or calcineurin inhibitors. The addition of systemic therapy is recommended for more satisfactory suppression of the disease. Although HCQ is a reasonable first‐line systemic therapy, it is often insufficient alone. Other agents such as methotrexate and mycophenolate mofetil can be added to the treatment regimen and have proven to be effective for both the cutaneous and muscle symptoms.^27^, ^28^, ^29^

In conclusion, NXP‐2 positive amyopathic DM can present only with cutaneous manifestations, without any classic features such as muscle weakness, dysphagia, and edema. These cutaneous manifestations can be nonspecific and heterogeneous, making them difficult to recognize early in the disease course as they can stray from typical DM findings. However, given the association of DM with a higher risk of malignancy and ILD, early recognition and accurate diagnosis are important especially since other clinical entities such as cutaneous lupus erythematosus can present similarly.

## AUTHOR CONTRIBUTIONS

**Divya M. Shan:** Writing – original draft; writing – review and editing. **Neha Gupta:** Writing – review and editing. **Alex G. Ortega‐Loayza:** Resources. **Sofia Shea:** Resources. **Abhishek Nandan:** Supervision; writing – review and editing.

## FUNDING INFORMATION

No funds, grants, or other support were received.

## CONFLICT OF INTEREST STATEMENT

All authors declare that they have no competing interests.

## CONSENT

Written and signed informed consent was obtained from the patient to publish this report in accordance with the journal's patient consent policy.

## Data Availability

Data sharing not applicable to this article as no datasets were generated or analysed during the current study.
